# Dose and time dependence of box jellyfish antivenom

**DOI:** 10.1186/1678-9199-20-34

**Published:** 2014-08-12

**Authors:** Athena Andreosso, Michael J Smout, Jamie E Seymour

**Affiliations:** 1 Australian Institute of Tropical Health and Medicine, Faculty of Medicine, Health and Molecular Sciences, James Cook University, McGregor Road, 4878 Cairns, Australia; 2 Centre for Biodiscovery and Molecular Development of Therapeutics, Australian Institute of Tropical Health and Medicine, Queensland Tropical Health Alliance Laboratory, James Cook University, McGregor Road, 4878 Cairns, Australia

**Keywords:** Chironex fleckeri, Antivenom, Venom, Dose–response curve

## Abstract

**Background:**

The effectiveness of the currently available box jellyfish (*Chironex fleckeri*) antivenom has been subject of debate for many years. To assess whether the box jellyfish antivenom has the ability to attenuate venom-induced damage at cellular level, the present study analyzed the dose and time dependence of the antivenom in a cell-based assay.

**Methods:**

Different doses of antivenom were added to venom and subsequently administered to cells and the cell index was measured using xCelligence Technology (ACEA Biosciences). Similarly, antivenom and venom were incubated over different time periods and cell survival measured as stated above. For both experiments, the cell index was plotted as a measure of cell survival against the dose or incubation time and significance was determined with the use of a one-way ANOVA with a LSD *post hoc* test.

**Results:**

Increasing concentrations of antivenom significantly augmented cell survival, with a concentration of approximately five times the currently recommended dose for human envenomation, causing the first significant increase in cell survival compared venom alone. Further, cell survival improved with increasing incubation time of venom and antivenom prior to addition to the cells, indicating that box jellyfish antivenom requires approximately 70 minutes to neutralize *C. fleckeri* venom.

**Conclusion:**

The presented results suggest that the currently recommended dose of antivenom requires adjustment, and more importantly, a human trial to test the effects of higher concentrations is also necessary. Further, antivenom has delayed neutralizing effects (i.e. after 70 minutes) which underlines the eminence of immediate and prolonged cardiopulmonary resuscitation in victims suffering from a *C. fleckeri* venom-induced cardiovascular collapse.

## Background

The Commonwealth Serum Laboratories™ (CSL) have been producing box jellyfish antivenom, raised from ovine antibodies since the 1970s [1]. Currently, *Chironex fleckeri* envenomation victims are commonly given CSL antivenom via intramuscular injection and there are several reports of pain amelioration as well as successful recovery of severely envenomed patients following its administration [2,3]. However, since *C. fleckeri* venom is distributed through the vascular system, it potentially leads to rapid cardiovascular collapse. Thus, intramuscular (*versus* intravenous) route has been criticized as an adequate via of administration [2]. In addition, there have been reports of severe envenomed victims dying despite (as well as some surviving without) the administration of antivenom, which question its effectiveness as well as its necessity [3-6].

Experimental evidence *in vitro* and *in vivo* also shows conflicting results. On one hand, it has been suggested that the venom extraction method influenced the effectiveness of antivenom. For example, the venom currently used for the serum production is obtained by an extraction method developed by Barnes [7] called “milking”. In this technique, jellyfish tentacles from *C. fleckeri* are placed on an amniotic membrane that is tensioned over a jar and through subsequent mechanical, chemical or sonic stimulation the nematocysts are prompted to deliver venom through the membrane into the collection jar [7]. Accordingly, prophylactic administration of antivenom significantly delayed death in mice injected with “milked” venom. However, the effects of one toxic agent in venom extracted directly from the nematocysts, i.e. “native” venom, could not be neutralized [8]. Conversely, antivenom raised from antibodies against both, milked and native venom, was able to prevent cardiovascular collapse in rats [9].

While it generally seems that CSL antivenom has the ability to attenuate venom effects, relatively high doses are required to achieve venom neutralization [9]. It has thus been suggested that the current initial treatment for human envenomation (one to three vials, i.e., 20,000-60,000 units) may be insufficient [9-11]. Further complications regarding the use and effectiveness of the antivenom exist with only prophylactic administrations being able to counteract the venom effects, which suggests that the antivenom is also too slow to attenuate the effects of *C. fleckeri* venom in a clinically relevant setting [10,12].

In the light of this, the present study analyses the dose and time dependence of CSL box jellyfish antivenom using a cell-based assay to create dose–response curves for antivenom *per se*, as well as for different venom-antivenom incubation times. Considering that currently there are no generally reliable treatments available, the results of the present study may have implications for the production of antivenom and possibly also for the current First Aid guidelines.

## Methods

### Venom extraction

Adult specimens of *C. fleckeri* were collected near Napranum, Qld, Australia (12.6835° S, 141.8884° E) in November 2011; tentacles were removed and nematocysts were collected according to Bloom *et al.*[13]. Venom extraction was carried out following the method of Carrette and Seymour [14]. In short, lyophilized nematocysts were put into 3-mL vials with approximately 5 mm (height) of glass-beads and filled with Milli-Q-water (MQ) (at 4°C). The mixture was then shaken at 5,000 rpm in a bead mill beater ten times for two minutes to maximize nematocyst rupture and venom yield. After each time in the beater the vials were placed in ice slurry for five minutes. Finally, the mixture was centrifuged for one minute (13,000 rpm); the supernatant (i.e. venom) was collected and subsequently lyophilized. The lyophilized venom was then stored at -80°C until required for use. Reconstituted venom was placed in slurry ice baths to preserve toxic activity of venom samples that were rehydrated for experimental procedures.

### Cell culture and Xcelligence system

Human cholangiocarcinoma cells (KKU-100 cell line, hereafter referred to as cancer cells) were cultured following the manufacturer’s instructions in 5 mL RPMI media (Gibco) with 10% fetal bovine serum at 37°C and 5% CO_2_ in 25 cm^2^ monolayer flasks. The cells were seeded and incubated in 96-E-well plates (ACEA Biosciences) at approximately 6,000 cells in 150 mL media per well for 24 hours at 37°C and 5% CO_2_ to allow the cells to attach to the bottom of the wells before treatment was applied. Cell adherence to the bottom of the plate, i.e. the cell index (CI), a measure of cell viability was monitored using xCELLigence system [15].

### Venom

Venom from *C. fleckeri* at a concentration of 1.47 μg/mL was used for all experiments as it induced approximately 70% death of cancer cells in ten minutes.

### Antivenom

Antivenom (batch: 0556–07301; expiry: 08/2015) concentrations were converted from volume concentration (μL/mL) to units per volume (units/mL) to allow for comparison with previous studies. Further, the currently recommended dose of one to three vials (i.e. 20,000-60,000 units) for one average adult (approx.70 kg) has been calculated into units per volume as follows. A 70 kg adult has a blood volume of approximately 4700 mL, of which only 55% is plasma (45% blood cells), thus one vial (20,000 units) of antivenom is administered to approximately 2585 mL (plasma volume) resulting in 7.7 units/mL (15.4 or 23.30 units/mL for two or three vials respectively) of antivenom in the vascular system (if given intravenously).

For the dose–response curve experiment, antivenom concentrations of 0–205.10 units/mL were used. Concerning incubation time, an antivenom concentration of 134.23 units/mL was incubated with venom (0–135 minutes), as this represented a concentration at which the venom had a clear effect on cell survival (50%). This allowed for all possible outcomes of venom-antivenom incubation effects: increased, unchanged and decreased cell survival.

### Dose–response curve and incubation time

For the dose–response curve, three replicates of each of the different concentrations of antivenom were mixed with the venom just before adding the mixture (20 μL) to the cells in the xCelligence plate. Additionally, a control with venom (but no antivenom), one with antivenom only and one with MQ-water (used for the dilutions) only were added to the cells in three replicates, each. The dose–response curve was then generated with the use of Prism (GraphPad Software), in brief, to analyze if there was a dose-dependent relationship, antivenom concentrations were plotted against cell survival.

Similarly, to test the effects of different venom-antivenom incubation times on cell survival three replicates of venom were incubated with antivenom over different periods of time (0, 2.5, 5, 10, 15, 20, 30, 40, 50, 60, 75 and 135 minutes) at 37°C (representing an approximate for the human body temperature). For this experiment, additionally to the above stated controls, three maximally (135 minutes) incubated replicates (20 μL) of each control were added to the cells. The data for nearly four hours post-treatment addition were then plotted and analyzed in Prism. All figures were normalized to the cell index of the control (MQ for the antivenom-dose–response curve and incubated MQ for incubation time experiment), which was set to 100% cell survival. For both experiments a one-way ANOVA with an LSD *post hoc* test was used to test for significant concentration- and incubation-time dependent increases in cell survival.

## Results

### Dose–response curve

**Figure 1:**
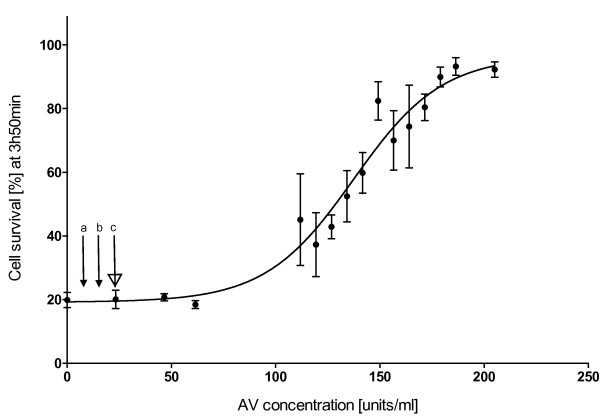
**Dose–response curve of *C. fleckeri *antivenom against a 1.47 μg/mL of venom for three hours and 50 minutes after exposure to the cells.** The error bars represent 95% CI for each mean (three replicates). The currently recommended dose for human envenomation – of one, two and three vials – is indicated (a, b and c, respectively) for reference.

Cell survival increased significantly with higher doses of antivenom (F_15,2_ = 38.901, p < 0.001) (Figure 1), thus clearly showing a dose-dependent relationship between antivenom concentrations and cell survival. The first concentration to have a significant effect on cell survival, relative to the control, was 134.24 units/mL, resulting in approximately 50% cell survival (LSD *post hoc*). This concentration is approximately a fivefold higher than the maximal recommended initial dose (three vials, i.e. 23.1 units/mL) for *C. fleckeri* envenomed humans. Concentrations higher than 180 units/mL resulted in nearly 100% survival of the cells, despite exposure to venom (1.47 μg/mL) and resulted in significantly higher cell survival than concentrations below 145 units/mL (LSD *post hoc*).

### Incubation time

**Figure 2:**
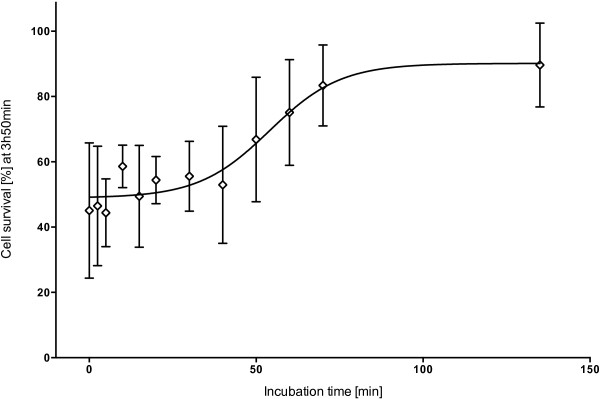
**Effect of incubation time of *C. fleckeri *antivenom (156.41 units/mL) with venom (1.47 μg/mL) on cell survival at three hours and 50 minutes after exposure to the cells.** The error bars represent 95% CI for each mean (three replicates).

Increasing incubation time had a significant effect on cell survival (F_12,2_ = 3.29, p = 0.008) (Figure 2). The first significant increase in cell survival was observed after a venom-antivenom incubation time of 70 minutes (LSD *post hoc*). Thus at a venom concentration of 1.47 μg/mL it took the antivenom (156.41 units/mL) approximately 70 minutes to effectively neutralize the venom effects, resulting in approximately 100% cell survival.

## Discussion

This is the first study presenting a dose–response curve for the commercially available CSL box jellyfish antivenom in a cell-based assay. The presented results clearly indicate that the effectiveness of the antivenom is dose-dependent, with higher concentrations of antivenom resulting in higher protective effects on cell survival. Similarly, a previous cell-based study using prophylactic antivenom concentrations between 0.04 and 5 units/mL on A7r5 cells (1 μg/mL of venom), found that concentrations below 5 units/mL are ineffective on cell survival [10]. Although the latter study used a different cell type, as well as lower venom and antivenom concentrations, the benefit of higher doses of antivenom is clear.

Similarly, *in vivo* studies reported that venom only (30 μg/kg, i.e. for an human adult approx. 2100 μg/70 kg, thus approx. 0.81 μg/mL blood plasma) premixed with 3,000 units/kg of antivenom (ca. 10.5 vials for a 70 kg adult) was able to inhibit cardiovascular collapse in anesthetized rats, whereas concentrations of 1,000 units/kg and 600 units/kg (ca. 3.5 and 2.1 vials per 70 kg adult) failed to do so [9]. These results slightly contradicted a previous study in which the same antivenom (3,000 units/kg) and venom dose (approx. 0.81 μg/mL) were used, yet, the majority of the anesthetized rats (60%) suffered fatal cardiovascular collapse [16]. However, with MgSO_4_ as adjunct treatment all test animals survived [16].

The present study used far higher antivenom concentrations and thereby venom deactivation was successful (with an antivenom dose of 180 units/mL, i.e. 23.5 vials/adult), thus suggesting that the currently recommended dose for human envenomation (three vials) is probably insufficient. Recurrent criticism as to the general ability of antivenom to reduce the effects of *C. fleckeri* toxins may well be justified, yet not due to the serum being unable to neutralize the venom as a recent immunoaffinity assay suggested. The study was originally designed to consider differences between antibodies raised against milked, i.e. CSL-antivenom, and native venom. It has demonstrated that compared to polyclonal rabbit IgG antibodies raised against native venom, the commercially available CSL-antivenom indeed contains all the necessary antibodies to the main toxic agents in *C. fleckeri* venom [9].

However, since CSL has announced their recommendation for an initial dose of one to three vials of antivenom for human envenomation, it has been discovered that *C. fleckeri* venom undergoes an ontogenetic shift from invertebrate- to vertebrate-specificity with adult (as opposed to young) animals being more toxic to humans [17]. Further, the geographic location is now known to also affect the toxicity of the venom [18]. Consequently, the age and collection location of the specimens used to obtain the venom to raise the antibodies may affect the potency of the antivenom. Additionally, accurate quantification of venom injected into a victim is improbable, since the magnitude of envenomation depends on the degree of tentacle contact, and thus conclusions on adequate doses of antivenom for human envenomation may be inaccurate.

In the present study, the period of incubation of *C. fleckeri* venom with antivenom also influenced its effectiveness regarding cell survival, which suggests that antivenom is not only dose- but also time-dependent. Considering that initial beachside emergency treatment for *C. fleckeri* envenomation currently recommends intramuscular administration of three vials (each 20,000 units/6.25 mL), this administration route may be slow and the dose small to attenuate venom effects. In most cases of severe *C. fleckeri* envenomation the venom is distributed through the vascular system, thus reaching its target tissue, the heart, within minutes [2]. In fatally envenomed victims death may occur in less than ten minutes (usually within 20 minutes) after contact with *C. fleckeri* tentacles [4,19-21].

In the present study, it took more than 60 minutes to antivenom to neutralize the venom. With intramuscular injection of antivenom, delivery of the treatment would be further inhibited, since it would also have to pass through the lymphatic system in order to reach the heart and inhibit cardiovascular collapse. Conversely, if intravenous injection of antivenom could be administered as soon as possible, and life saving measures such as cardiopulmonary resuscitation could be undertaken unceasingly (as required by the victim), the antivenom would show its beneficial effects earlier. However, this partially implies that the effects of the venom are reversible (including that venom already bound to cells can be neutralized), which may contradict the currently believed pore-forming mode of action [2,12,22]. Finally, in the light of the rapid onset of symptoms and the fact that experimentally only prophylactic doses of antivenom were effective, the window of opportunity for antivenom administration may indeed be too short [16,19]. However, this cannot be assumed until higher initial doses have been tried.

## Conclusion

The present study is the first to create a dose–response curve and to compare different incubation times for CSL antivenom and all evidence suggests that the potential for venom neutralization is absolutely apparent. However, current recommended doses (three vials) seem far too small and thus need to be adjusted. At higher doses, antivenom appeared to also improve its neutralizing ability with incubation time, thus suggesting that the venom-antivenom binding may be a process ongoing for just about over one hour. Thus, chances for successful treatment following a *C. fleckeri* envenomation may improve if the concentration of antivenom as well as the given dose are increased and the life saving measures are exceptionally prolonged in order to provide time for the antivenom act on the venom.

## Competing interests

The authors declare that there are no competing interests.

## Authors’ contributions

AA wrote the first draft of the manuscript and performed cell culture and laboratory work. JS, MS and AA contributed to the experimental design, analyzed data and contributed to writing and editing of the manuscript. All authors read and approved the final manuscript.
